# Abernethy Malformation Masquerading as Congenital Heart Disease: A Boy With Cyanosis, Clubbing, and Hypoxia

**DOI:** 10.7759/cureus.33519

**Published:** 2023-01-08

**Authors:** Rohit Agarwal, Durga Prasad, Gaurav Chauhan, Abhai Verma

**Affiliations:** 1 Interventional Radiology, Medanta Medicity, Lucknow, IND; 2 Pediatric Gastroenterology, Medanta Medicity, Lucknow, IND; 3 Radiology, Medanta Hospital, Lucknow, IND; 4 Gastroenterology, Medanta Hospital, Lucknow, IND

**Keywords:** abernethy malformation, portosystemic shunt, congenital anomalies of the liver, severe dyspnea, congenital extracardiac portosystemic shunt, vascular plug

## Abstract

Abernethy malformation is an extrahepatic congenital portosystemic shunt characterized by the diversion of the portal blood away from the liver through a shunt that drains directly into the inferior vena cava. We present a case of a male child with Abernethy malformation, which was initially diagnosed as cyanotic heart disease due to pulmonary arteriovenous malformation. However, after proper clinical evaluation and investigations, the diagnosis of Abernethy malformation was established. Thereafter, the patient was successfully treated with endovascular embolization. At one year follow-up, marked relief in exertional dyspnea and improvement in physical growth was achieved with no observable complications.

## Introduction

Abernethy malformation is an extrahepatic congenital portosystemic shunt (CPSS), also known as congenital extrahepatic portosystemic shunt (CEPS), which is an extremely rare condition characterized by the diversion of portal blood away from the liver. In this anomaly, blood from splanchnic circulation drains into the inferior vena cava (IVC) through a shunt bypassing the liver, thereby causing an alteration in the metabolism of pulmonary vasoactive substances resulting in pulmonary vasodilatation, diffusion-perfusion mismatch, and eventually hypoxemia [1-3]. The clinical manifestations of Abernethy malformation are highly variable and can be asymptomatic or symptomatic due to the shunting of blood such as hepatic encephalopathy or hepatopulmonary syndrome (HPS). Abernethy malformation can be classified into two types. Type I is defined by an absence of intrahepatic portal veins (PV), and a lack of liver perfusion with portal blood. In type Ia, superior mesenteric and splenic veins drain separately into the IVC, and in type Ib splanchnic blood drain via the common trunk into IVC [4]. Type II is defined by side-to-side anastomosis of the PV with IVC in which the hypoplastic intrahepatic PV supplies some degree of portal flow to the liver parenchyma [4]. Prolonged untreated severe hypoxemia can result in irreversible changes in the pulmonary vasculature and its consequences become incapacitating for the patient. Early shunt closure resolves hypoxemia and clinical symptomatology.

## Case presentation

We present a case of a nine-year-old boy from a rural background with a height of 112 cm (<3rd centile) and weighing 19 kg (<3rd centile), who presented with cyanosis, clubbing, dyspnea, and restricted growth since four years of age. Physical examination on admission revealed central cyanosis and digital clubbing with resting pulse oximetry (SpO2) of 60% (normal range > 95%) on room air. Blood investigations showed an elevated hemoglobin level of 15.5 g/L (normal range: 11.5-15 g/L) and a normal liver enzyme profile with aspartate aminotransferase (AST) of 32 U/L (normal range: 17-59 U/L) and alanine aminotransferase (ALT) of 19 U/L (normal range: 21-72 U/L). He had an elevated serum ammonia level of 77 μmol/L (normal range: 7-30 μmol/L) but no evidence of hepatic encephalopathy was found. His coagulation profile was slightly deranged with an international normalized ratio (INR) of 1.39 (normal range: 0.8 to 1.1) and activated partial thromboplastin time (APTT) of 30 seconds (normal range: 24.6-29.9 seconds).

**Figure 1:**
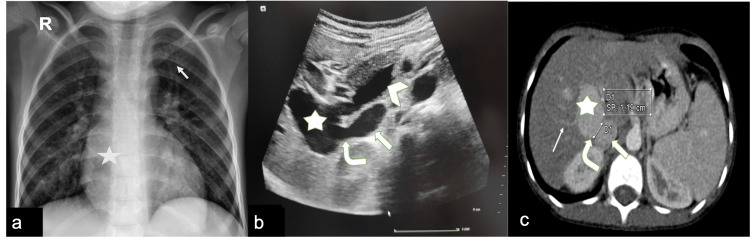
(A) Chest X-ray showing mild cardiomegaly (star) and prominent vascular marking (arrow). (B) Ultrasonography image showing side-to-side communication (curved arrow) between the main portal vein (star) and inferior vena cava (solid arrow) and also the patent superior mesenteric vein (arrowhead). (C) Corresponding CT scan image at a similar level showing side-to-side communication (curved solid arrow) between the main portal vein (star) and inferior vena cava (solid arrow), shunt size (line), and also the patent hypoplastic intrahepatic portal venous branches (simple arrow).

Chest X-ray showed mild cardiomegaly with increased pulmonary vascular markings (Figure 1A). Electrocardiogram (ECG) was unremarkable. Echocardiography revealed features of mild left-sided volume overload and dilated left atrium, after that, a contrast echocardiography was performed with agitated saline, which showed the appearance of micro-bubbles in the left atrium after four cardiac cycles, suggesting pulmonary arteriovenous communication.

Doppler ultrasound showed dilated extrahepatic main PV with hypoplastic intrahepatic portal venous branches arising from the dilated segment and a large communicating shunt between IVC and the main PV (Figure 1B). Computed tomography (CT) angiography confirmed the Doppler findings and a diagnosis of type II Abernethy malformation was made (Figure 1C). Hepatic veins were normal on Doppler and CT angiography. Once the diagnosis was made, the patient was counseled regarding the treatment option.

**Figure 2:**
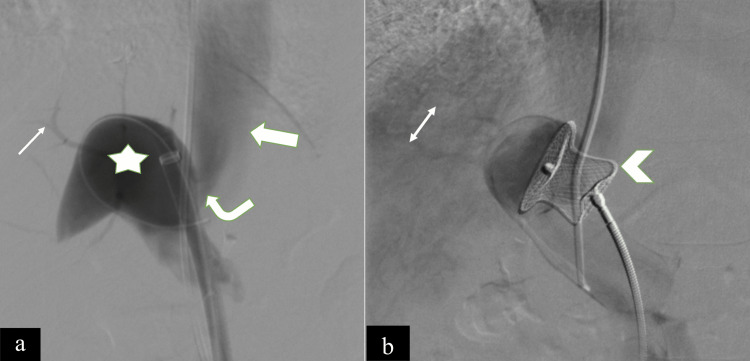
(A) Digital subtraction angiogram showing side-to-side communication (curved bold arrow) between the main portal vein (star) and inferior vena cava (solid arrow) along with patent hypoplastic intrahepatic portal venous branches (simple arrow). (B) Digital subtraction angiogram showing post-deployment of the vascular plug (arrowhead) showing stasis and hepatic parenchymal blush (double arrow).

The patient was taken under general anesthesia for endovascular embolization of CPSS. Both jugular and femoral access were taken. The shunt was cannulated from both sides (jugular and femoral). Multiple catheter angiograms were taken to establish the patency of the intrahepatic portal venous branches (Figure 2A). After documenting the patency of intrahepatic portal venous branches, the decision of shunt closure was taken. Angiogram showed the diameter of the shunt as 12 mm with the length of the shunt being 7 mm. After proper evaluation, the decision was taken to close the shunt with a vascular plug closure device (Cera, LifeTech Scientific Corporation, Shenzhen, China). The device was deployed across the shunt via the femoral route and angiograms were taken from the jugular route, which showed a visualization of the intrahepatic portal branches and immediate closure of the shunt (Figure 2B).

## Discussion

In the presence of cyanosis and clubbing, clinicians usually think about congenital heart disease until proven otherwise; however, there are uncommon conditions like hemoglobinopathy, Abernethy malformation, and diffuse pulmonary arteriovenous fistula that can also result in cyanosis. Most of these patients end up in cardiothoracic surgery or cardiology clinics. Hence, special attention and detailed workup should be paid to children with unexplained cyanosis where cardiac causes have been ruled out. Similarly, our case was misdiagnosed, mistaken, and managed as cyanotic heart disease for many years before coming to our hospital. In resource-poor settings, the diagnosis of CPSS can be missed or delayed due to a lack of resources and technical expertise. However, nowadays, it can be picked up easily due to advancements in imaging techniques. Diagnosis of CPSS is often delayed and difficult due to varied clinical presentations. These patients reach the healthcare facility when complications have set in. Abernethy malformation may lead to severe complications such as liver tumors, hepatic encephalopathy, HPS, or pulmonary hypertension. HPS is one of the common complications and has been reported in at least 20 children with Abernethy malformation [5]. HPS is a triad of liver diseases, arterial hypoxemia, and pulmonary vascular dilatation. Although HPS typically develops in the setting of cirrhosis and portal hypertension, it may also occur in the absence of parenchymal liver disease in association with portosystemic shunting. It is believed to be attributed to the exposure of the pulmonary vascular bed to vasoactive mediators, which are derived from the intestinal tract entering the systemic circulation without being metabolized in the liver [3]. Prolonged untreated cases result in pulmonary vasodilatation, pulmonary arteriovenous fistula, diffusion-perfusion mismatch, and eventually hypoxemia.

Treatment of CPSS depends on the type, the presenting symptoms, complications, and comorbidity. Treatment may vary from endovascular occlusion of the shunt and surgical correction to even liver transplantation [5,6]. The optimal timing of the shunt closure is not defined in pediatric patients with CPSS [7]. It is proposed that even in the absence of overt symptoms, early intervention prevents pulmonary and other complications [8].

Liver transplantation is the treatment of choice for Abernethy malformation type I due to the lack of an apparent intrahepatic portal venous channel. But recently published experiences by several authors point out that many patients with CPSS type I malformations might have small portal vein radicals, which cannot be seen on ultrasonography but could be visualized on shunt angiography [9]. The balloon occlusion test of the fistula can also be done. This test helps to decide on a single-stage or a two-stage shunt closure procedure.

Transcatheter occlusion/surgical ligation of the shunt is the therapeutic option for type II* *Abernethy malformation [9]. The successful transcatheter closure of CPSS has been conducted in children with type II Abernethy malformation with good outcomes [9]. In general, exercise tolerance and resting oxygen saturation of these patients is increased after the procedure [5]. However, symptoms like exertional dyspnea, cyanosis, and pulmonary hypertension show partial improvement when the treatment is delayed [5]. Shunt closure results in the restoration of intrahepatic portal blood flow in most patients. Symptomatic improvement and stabilization of pulmonary, cardiac, and neurological functions are seen in patients post-shunt closure.

In our case, device closure of the shunt was successful, as his serum ammonia levels normalized over one month and hypoxemia resolved over six months. At one year follow-up, the child's growth and exercise tolerance improved, and resting oxygen saturation increased from 60% to 92% on room air. There were no complications observed at the one-year follow-up post-shunt occlusion. Early diagnosis and appropriate management of Abernethy malformation may lead to an improved prognosis.

## Conclusions

In conclusion, Abernethy malformation is a rare cause of cyanosis and clubbing in children. Clinicians must suspect Abernethy malformation complicated with HPS in children in the setting of unexplained cyanosis where common causes of cyanosis and dyspnea have been ruled out. Mere knowledge of the disease entity and high clinical suspicion can lead to early diagnosis and possible treatment. The treatment options range from complex liver transplantation to simple endovascular closure of the shunt depending upon the type of shunt and underlying liver abnormality.

## References

[1] Abernethy J (1797). Account of two instances of uncommon formation in the viscera of the human body: from the philosophical transactions of the Royal Society of London. Med Facts Obs.

[2] Hao Y, Hong X, Zhao X (2015). Congenital absence of the portal vein associated with focal nodular hyperplasia of the liver and congenital heart disease (Abernethy malformation): a case report and literature review. Oncol Lett.

[3] Sokollik C, Bandsma RH, Gana JC, van den Heuvel M, Ling SC (2013). Congenital portosystemic shunt: characterization of a multisystem disease. J Pediatr Gastroenterol Nutr.

[4] Morgan G, Superina R (1994). Congenital absence of the portal vein: two cases and a proposed classification system for portasystemic vascular anomalies. J Pediatr Surg.

[5] Fu L, Wang Q, Wu J (2016). Congenital extrahepatic portosystemic shunt: an underdiagnosed but treatable cause of hepatopulmonary syndrome. Eur J Pediatr.

[6] Singhal A, Srivastava A, Goyal N, Vij V, Wadhawan M, Bera M, Gupta S (2009). Successful living donor liver transplant in a child with Abernethy malformation with biliary atresia, ventricular septal defect and intrapulmonary shunting. Pediatr Transplant.

[7] Papamichail M, Pizanias M, Heaton N (2018). Congenital portosystemic venous shunt. Eur J Pediatr.

[8] Papamichail M, Ali A, Quaglia A, Karani J, Heaton N (2016). Liver resection for the treatment of a congenital intrahepatic portosystemic venous shunt. Hepatobiliary Pancreat Dis Int.

[9] Franchi-Abella S, Branchereau S, Lambert V (2010). Complications of congenital portosystemic shunts in children: therapeutic options and outcomes. J Pediatr Gastroenterol Nutr.

